# Differences in the Expression and Distribution of Flotillin-2 in Chick, Mice and Human Muscle Cells

**DOI:** 10.1371/journal.pone.0103990

**Published:** 2014-08-08

**Authors:** Ana Claudia Batista Possidonio, Carolina Pontes Soares, Débora Morueco Portilho, Victor Midlej, Marlene Benchimol, Gillian Butler-Browne, Manoel Luis Costa, Claudia Mermelstein

**Affiliations:** 1 Laboratório de Diferenciação Muscular e Citoesqueleto, Instituto de Ciências Biomédicas, Universidade Federal do Rio de Janeiro, Rio de Janeiro, Brazil; 2 Université Pierre et Marie Curie Paris 6, Paris, France; 3 Laboratório de Ultraestrutura Celular, Universidade Santa Úrsula, Rio de Janeiro, Brazil; Institut de Myologie, France

## Abstract

Myoblasts undergo a series of changes in the composition and dynamics of their plasma membranes during the initial steps of skeletal muscle differentiation. These changes are crucial requirements for myoblast fusion and allow the formation of striated muscle fibers. Membrane microdomains, or lipid rafts, have been implicated in myoblast fusion. Flotillins are scaffold proteins that are essential for the formation and dynamics of lipid rafts. Flotillins have been widely studied over the last few years, but still little is known about their role during skeletal muscle differentiation. In the present study, we analyzed the expression and distribution of flotillin-2 in chick, mice and human muscle cells grown *in vitro*. Primary cultures of chick myogenic cells showed a decrease in the expression of flotillin-2 during the first 72 hours of muscle differentiation. Interestingly, flotillin-2 was found to be highly expressed in chick myogenic fibroblasts and weakly expressed in chick myoblasts and multinucleated myotubes. Flotillin-2 was distributed in vesicle-like structures within the cytoplasm of chick myogenic fibroblasts, in the mouse C2C12 myogenic cell line, and in neonatal human muscle cells. Cryo-immunogold labeling revealed the presence of flotillin-2 in vesicles and in Golgi stacks in chick myogenic fibroblasts. Further, brefeldin A induced a major reduction in the number of flotillin-2 containing vesicles which correlates to a decrease in myoblast fusion. These results suggest the involvement of flotillin-2 during the initial steps of skeletal myogenesis.

## Introduction

The formation of a mature skeletal muscle fiber depends on a series of events that includes the proliferation of myoblasts, their withdrawal from the cell cycle, cell migration, cell recognition and cell adhesion followed by membrane fusion, culminating with the formation of long and striated multinucleated myotubes. All these events can be reproduced *in vitro* in cultures of cells isolated from embryonic muscle tissues. One of the most robust myogenic cell culture models is that obtained from embryonic chick pectoral muscle. Although myoblast fusion has been extensively studied over the last fifty years, this complex cellular process is still not completely understood. Many membrane components have been implicated in the recognition and fusion of myoblasts, such as cadherins [1], cholesterol [2], and phosphatidylserine [3]. It has been shown that cholesterol-enriched membrane microdomains, or lipid rafts, play a role in myoblast adhesion and fusion [4]. Two different types of membrane microdomains can be found in eukaryotic cells: planar and caveolar rafts. Planar rafts are characterized by the presence of flotillin proteins while caveolin proteins are present in caveolar rafts. Flotillins and caveolins are scaffold proteins that are involved in the formation and function of membrane microdomains. The two types of flotillins that can be found in planar rafts, flotillin-1 and flotillin-2, are products of different genes; both have a molecular weight of 48 kDa and they share 44% identity in their primary sequence [5]. In addition to their prominent localization at the plasma membrane, both flotillins reside in intracellular compartments [6], where they localize to lipid droplets [7] and to compartments of the endocytic pathway, such as recycling endosomes [8]. Flotillins are involved in many cellular functions, including endocytosis, exocytosis, membrane cycling, formation and maintenance of lipid rafts in the membrane, cell signaling, cell migration and cell adhesion. Although flotillins have been widely studied over the last years in several cell types, still little is known about their involvement in skeletal muscle development. Interestingly, it has been shown that mature mouse skeletal muscles (diaphragm and psoas) are the main source of flotillin-1 while flotillin-2 is virtually absent in these mature muscles [9]. In the present work, we studied the expression and distribution of flotillin-2 in chick, mouse and human muscle cells grown *in vitro*. Our results show that flotillin-2 appears in vesicle-like structures within the cytoplasm of mouse C2C12 cells, human muscle cells, and chick myogenic fibroblasts. In chick myogenic cultures, flotillin-2 was found to be highly expressed in chick fibroblasts, barely expressed in myoblasts and almost excluded from multinucleated myotubes. Our data also show a down-regulation of flotillin-2 during chick skeletal muscle differentiation and suggest the involvement of flotillin-2 during the initial steps of avian myogenesis.

## Materials and Methods

### Antibodies and probes

DNA-binding probe DAPI (4,6-Diamino-2-phenylindole dyhydrochloride) was obtained from Molecular Probes (USA). Mouse monoclonal anti-flotillin-2 antibody (clone 29) was from BD Transduction Labs (USA). Rabbit polyclonal anti-chick desmin antibody was a gift from Dr. Howard Holtzer (University of Pennsylvania, USA). Rabbit anti-chick MyoD antibody [10] was kindly provided by Dr. Bruce Paterson (National Cancer Institute, NIH, USA). Mouse monoclonal anti-α-tubulin (clone DM1A) was from Sigma-Aldrich (USA). Alexa Fluor 488-goat anti-mouse/rabbit IgG and Alexa Fluor 546-goat anti-mouse/rabbit IgG antibodies were from Molecular Probes (USA). Peroxidase-conjugated goat anti-mouse/rabbit antibodies were obtained from Amersham Biosciences (UK). Goat anti-mouse 10-*η*m gold-conjugated antibody was from BB International (USA).

### Cell cultures

This study using chick embryos was approved by the Ethics Committee for Animal Care and Use in Scientific Research from the Federal University of Rio de Janeiro and received the approval number: DAHEICB 004, which approved the complete animal part of the study. All cell culture reagents were purchased from Invitrogen (São Paulo, Brazil). Primary cultures of myogenic cells were prepared from breast muscles of 11-day-old chick embryos [2]. Cells were grown in 2 ml of medium (Minimum Essential Medium with the addition of 10% horse serum, 0.5% chick embryo extract, 1% L-glutamine and 1% penicillin-streptomycin) at an initial density of 7.5×10^5^ cells/35 mm culture dishes on 22 mm-Aclar plastic coverslips (Pro-Plastics Inc., USA) previously coated with rat tail collagen. Cells were grown in a humidified 5% CO_2_ atmosphere at 37°C and were fed daily with fresh cultured medium.

The percentage of myoblasts in these chick myogenic cell cultures was calculated by the double-labeling of 24-hour cultures with both DAPI (nuclear staining) and anti-desmin antibody (a muscle-specific marker) [11] and subsequently counting the number of desmin-positive mononucleated cells out of the total number of cells in the field. On average, myoblasts made up 80% of each culture and fibroblasts comprised 20%.

Some 24-hour myogenic chick cultures were treated with the cholesterol depleting agent methyl-β-cyclodextrin (MbCD, Sigma) at a final concentration of 2 mM for 30 min. After treatment, cells were grown in fresh culture medium. The 2 mM final concentration of MbCD was chosen for cell culture treatment because our group has previously shown that 2 mM of MbCD is sufficient to induce chick skeletal muscle cell differentiation without interfering with cell viability [2].

Some 24-hour myogenic chick cultures were treated with brefeldin A (Sigma) at a final concentration of 5 ηg/ml for 3 hours or treated with nocodazole (Sigma) at a final concentration of 10 ηg/ml for 3 hours. After treatment, cells were grown in fresh culture medium for the next 3 hours.

Myoblast fusion index was quantified in untreated and treated chick myogenic cultures. The fusion index is defined as the number of nuclei in myotubes divided by the total number of nuclei. Cells were fixed with paraformaldehyde and double-labeled for desmin and DAPI. Nuclei were counted in fifty randomly chosen microscope fields (3 culture dishes for each of the experimental condition, 3 fields in each dish) at a magnification of ×40.

Mouse C2C12 cells (ATCC, USA) were grown in Dulbecco's modified Eagle's medium (DMEM) containing 10% fetal bovine serum and 1% penicillin-streptomycin.

Neonatal human myoblasts were isolated from the quadriceps muscle of a healthy 5-day-old infant with no signs of neuromuscular disorder [12] in accordance with the French legislation on ethical rules. Permission was received from the local ethical committee of Saint Vincent de Paul's children's hospital in Paris (France). Written and approved consent was obtained from the parents prior to autopsy when the quadriceps muscle biopsy was taken. Myoblasts were cultivated in proliferation medium which consisted in DMEM supplemented with 50 ηg/ml of gentamycin (Invitrogen, France) and 20% fetal bovine serum (Invitrogen, France) and incubated at 37°C in a humid air atmosphere containing 5% CO_2_. In all experiments, the minimal myogenic purity was 80% for neonatal cells. To induce differentiation, when cells were 90% confluent, the proliferation medium was replaced by DMEM supplemented with 10 ηg/ml of insulin (Sigma) and 100 ηg/ml of transferrin (Sigma).

### Immunofluorescence and digital image acquisition

Cells were rinsed with Phosphate Buffered Saline (PBS) and fixed with either 4% paraformaldehyde in PBS for 10 min at room temperature or with methanol for 10 min at 4°C. They were then permeabilized with 0.5% Triton-X 100 in PBS 3 times for 10 min. The same solution was used for all subsequent washing steps. Cells were incubated with primary antibodies for 1 h at 37°C. After incubation, cells were washed for 30 min and incubated with Alexa Fluor-conjugated secondary antibodies for 1 h at 37°C, and nuclei were labeled with DAPI (0.1 ηg/ml in 0.9% NaCl). Cells were mounted in Prolong Gold solution (Invitrogene) and examined with an Axiovert 100 microscope (Carl Zeiss, Germany) or with a laser scanning confocal microscope (TCS SP5 AOBS, Leica, Japan). Image processing and stack projections were performed using Fiji software (based on ImageJ, http://imageJ.nih.gov/ij/). Control experiments with no primary antibodies showed only a faint background staining (data not shown). Some cultured cells were also examined under phase contrast microscopy with an Axiovert 100 microscope (Carl Zeiss, Germany).

### SDS-PAGE and immunoblotting

Chick myogenic cells were grown for 24, 48 and 72 hours. Cultures were then quickly washed in ice-cold PBS. Fifty ηL of ice-cold sample buffer (4% SDS, 20% glycerol, 0.2 M dithiothreitol, 125 mM Tris-HCl pH 6.8) were added to the cultures and then cells were scraped off the dish with a plastic cell scraper. Cell extracts were recovered in a tube, centrifuged, and boiled for 5 min. The amount of protein in each sample was determined according to the Bradford method [13], using bovine serum albumin as a standard. Equal amounts of protein were loaded on 10% SDS-polyacrylamide gels prior to electrophoresis (SDS-PAGE). Proteins were then transferred to PVDF membranes. The proteins immobilized on the membranes were immediately blocked for 1 hour at room temperature in 5% non-fat dried milk in Tris buffered saline-Tween 20 solution (0.001%) (TBS-T). The membranes were then incubated for 12 hours at 37°C with an anti-flotillin-2 antibody. After 5 washes in TBS-T (3 min each), the membranes were incubated for 1 hour at 37°C with an anti-mouse peroxidase conjugated antibody (Amersham, dilution 1∶10,000 in TBS-T), washed again as described above and the bands were visualized using ECL plus Western Blotting Detection System (Amersham). To check sample loading, other membranes with the same samples were incubated with a mouse monoclonal anti-α-tubulin antibody (Sigma, dilution 1∶3000 in TBS-T-milk). After 5 washes in TBS-T (3 min each), membranes were incubated with anti-mouse peroxidase conjugated antibody (Amersham, dilution 1∶10,000 in TBS-T) and developed as described above. Quantification of protein bands was performed using the public domain software ImageJ (http://rsb.info.nih.gov/ij/) with data obtained from three independent experiments.

### Transmission electron microscopy (TEM)

Chick myogenic cells were grown for 48 hours and fixed overnight at room temperature in 2.5% (v/v) glutaraldehyde in 0.1 M cacodylate buffer, pH 7.2. Afterwards, the cells were washed three times in PBS and post-fixed for 40 min in 1% OsO4 in 0.1 M cacodylate buffer containing 5 mM CaCl_2_ and 0.8% potassium ferricyanide. The cells were dehydrated in acetone and embedded in Epon. Cells were fixed and processed *in situ* in the same culture dish where they were grown. All the subsequent steps were performed with the cells still in the culture dish. After embedding, a thin layer of the embedded cells was cut and added to a new empty Epon bloc. Ultra-thin sections 70–90 *η*m thick were harvested on 300-mesh copper grids, stained with 5% uranyl acetate and 1% lead citrate, and then observed with a JEOL 1210 transmission electron microscope (Japan).

### Cryo-immunogold electron microscopy

Chick myogenic cells were grown for 48 hours and fixed in 4% paraformaldehyde and 0.1% glutaraldehyde diluted in 0.1 M sodium cacodylate buffer (pH 7.2) for 1 h at 4°C in a 35 mm culture dish. The cells were harvested with a cell scraper, washed twice in PBS, embedded in 2.3 M sucrose, and frozen in liquid nitrogen. Thin cryosections were obtained with a Leica EM FCS cryoultramicrotome (Germany). The sections were blocked in 50 mM NH_4_Cl and 3% bovine serum albumin in PBS, pH 8.0 for 30 min, and incubated with a monoclonal anti-flotillin-2 antibody (BD Transduction Laboratories, USA) diluted at 1∶5, followed by a goat anti-mouse 10-*η*m gold-conjugated (BB International, USA) diluted at 1∶100. The samples were processed according to Tokuyasu [14] and analyzed with a JEOL 1210 transmission electron microscope (Japan).

### RT-PCR analysis

Total RNA was extracted using TRIzol (Invitrogen, France) following the manufacturer's protocol. cDNA was synthesized from 1 ηg total RNA with reverse transcription (SuperScriptIII; Invitrogen) using oligo (dT) primer (Invitrogen). Quantitative PCR was performed on LightCycler 480 Real-Time PCR System (Roche, France) using a SYBR green PCR kit (Applied Biosystems, France), according to the manufacturer's protocol. Cycling conditions were as follows: initial denaturation step at 95°C for 10 min, followed by 40 cycles of 10 seconds at 95°C and 45 seconds at 60°C. Relative expression was calculated using the comparative Ct method. The GAPDH signal was used for normalization. PCR products were analyzed on 2% agarose gels, stained by ethidium bromide.

Primer sequences are listed below:

NM_001030719.1 *Gallus gallus* flotillin 2 (FLOT2), mRNA

product length = 74

Forward primer 1 GGCCTACGAGCTTCAAAGTG 20

Reverse primer 1 GTTGCACCACCTCGATTTCT 20

NM_204305.1 *Gallus gallus* glyceraldehyde-3-phosphate dehydrogenase (GAPDH), mRNA

product length = 112

Forward primer 1 GACGTGCAGCAGGAACACTA 20

Reverse primer 1 CTTGGACTTTGCCAGAGAGG 20

### Statistical analysis

Data are expressed as mean ± standard error of the mean (SEM) and represent one of at least three separate experiments performed in triplicate. Significance was determined using one-way analysis of variance (ANOVA) followed by Tukey post hoc test or the t test for unpaired samples. Differences <0.05 were considered significant.

## Results and Discussion

Myogenesis is a multistep developmental program that generates skeletal muscles. Myoblast proliferation, migration and fusion are the major steps that culminate with the formation of multinucleated contractile fibers. Changes in the structure and function of the plasma membrane occur during myogenesis. In this work we analyzed the expression and distribution of flotillin-2 in chick, mouse and human muscle cells grown *in vitro*.

**Figure 1:**
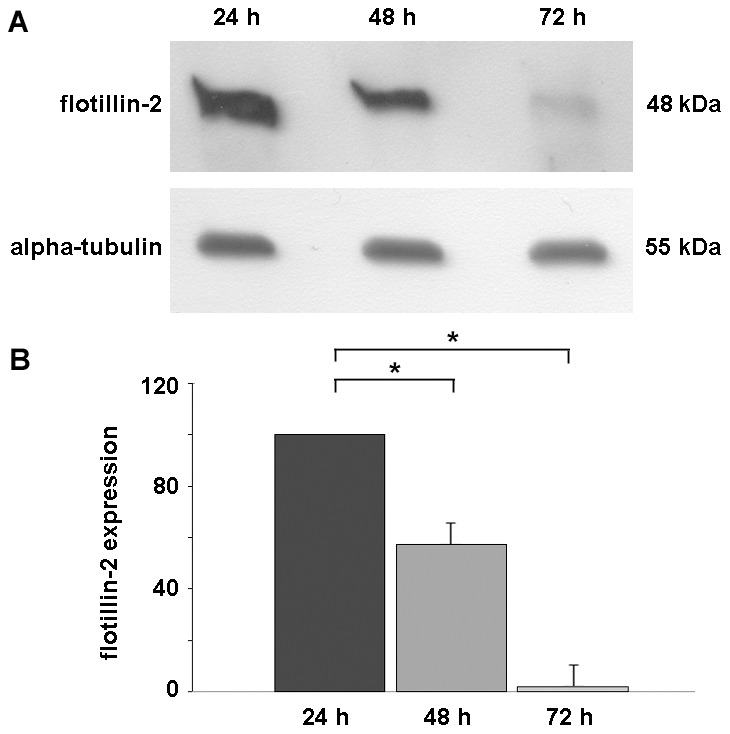
Flotillin-2 is down-regulated during *in vitro* chick skeletal myogenesis. Chick myogenic cells were grown for 24, 48 and 72-2. (**A**) Upper Western blot shows flotillin-2 reactivity and lower Western blot shows α-tubulin reactivity of the same samples, and was used to normalize sample loading. (**B**) Quantification of protein bands revealed a progressive decrease in the levels of flotillin-2 expression during skeletal muscle differentiation. *p<0.05; ANOVA followed by Tukey post hoc test versus 24-h group, n = 3.

First, we analyzed the expression of flotillin-2 protein during chick *in vitro* myogenic differentiation. Chick myogenic cells were grown for 24, 48 and 72 hours, and cell culture extracts were analyzed by Western blot using an antibody against flotillin-2. Figure 1 shows that flotillin-2 is highly expressed in the first 24–48 hours of culture, and it is barely detectable in 72-h cultures. Chick myogenic cultures at 24 h are mainly composed of mononucleated cells, namely fibroblasts and myoblasts, while 48-h cultures contain fibroblasts, myoblasts and young multinucleated myotubes. Seventy-two-hour cultures are mainly composed of mature multinucleated myotubes (thicker and longer than those found in 48-h cultures), with a reduced number of fibroblasts and myoblasts. These results show that flotillin-2 is down-regulated during chick *in vitro* myogenesis and suggest the involvement of flotillin-2 in the initial steps of skeletal muscle differentiation. In contrast with these results obtained on chick muscle cells, it has been described previously that flotillin-2 is up-regulated during the differentiation of the mouse cell line C2C12 [9]. There are at least two possible explanations for these contradictory results: (i) differences between chick and mouse; and/or (ii) differences between primary cultures and immortalized cell lines. Primary cultures of chick skeletal muscle cells behave very differently from muscle cell lines (such as C2C12). Differentiation of muscle cell lines must be induced by external stimuli and the most common way is the reduction of serum in the culture medium. In contrast, chick primary myoblast cultures do not depend on variations in the amount of serum to differentiate. Skeletal myogenesis occurs in a robust and autonomous way in primary chick myoblast cultures, culminating with the formation of myotubes that can contain more than one hundred nuclei per cell.

**Figure 2:**
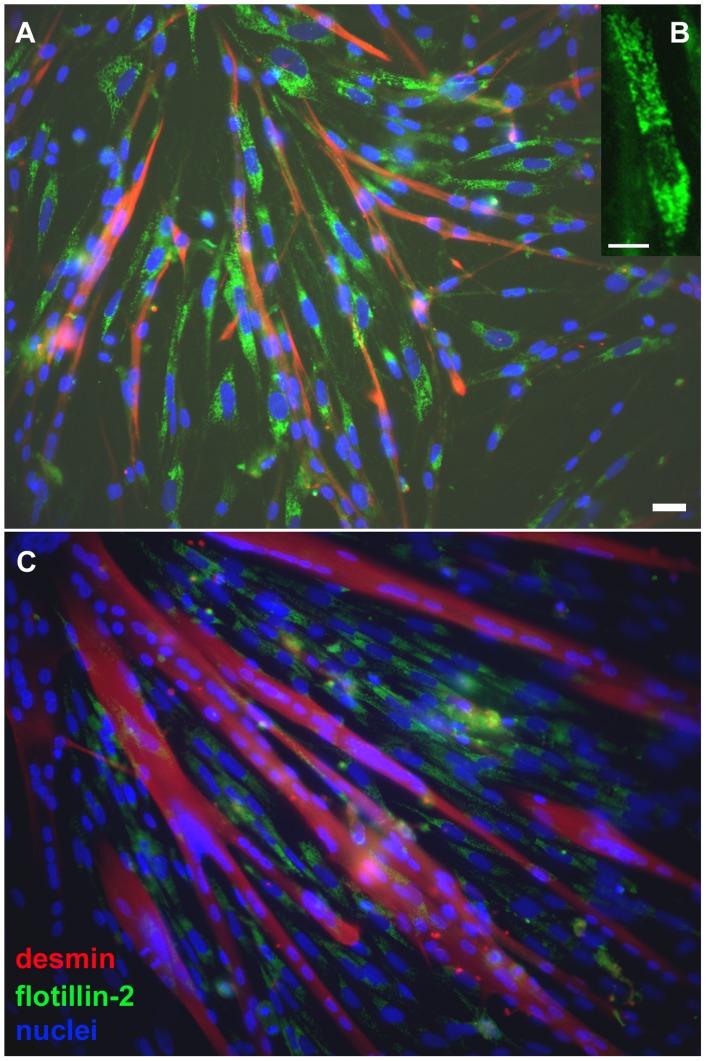
Flotillin-2 distribution in myogenic cells during skeletal muscle differentiation. Myogenic cells were grown for 24(**A** and **B**) or 72 hours (**C**). Cells were fixed with paraformaldehyde and stained with antibodies against desmin (**red**, **A** and **C**) and flotillin-2 (**green**, **A, B** and **C**) and with the nuclear dye DAPI (**blue**, **A** and **C**). Merged images are shown in **A** and **C**. Note that with paraformaldehyde fixation it is possible to see flotillin-2 almost exclusively in mononucleated cells in vesicle-like structures (**B**) and nearly absent from myotubes (**A** and **C**). Scale bar in **A** and **C** represents 20 µm and in **B** represents 10 µm.

Since chick primary myogenic cultures contain fibroblasts, myoblasts and multinucleated myotubes, we examined the distribution of flotillin-2 in the different types of cells. Primary cultures of chick skeletal muscle cells were grown for 24 and 72 hours, fixed with paraformaldehyde and immunolabeled for flotillin-2. Flotillin-2 was found predominantly in mononucleated cells and, interestingly, was almost absent from multinucleated myotubes (Figure 2). It is important to point out that mononucleated cells comprise both fibroblasts and myoblasts in chick primary myogenic cultures.

**Figure 3:**
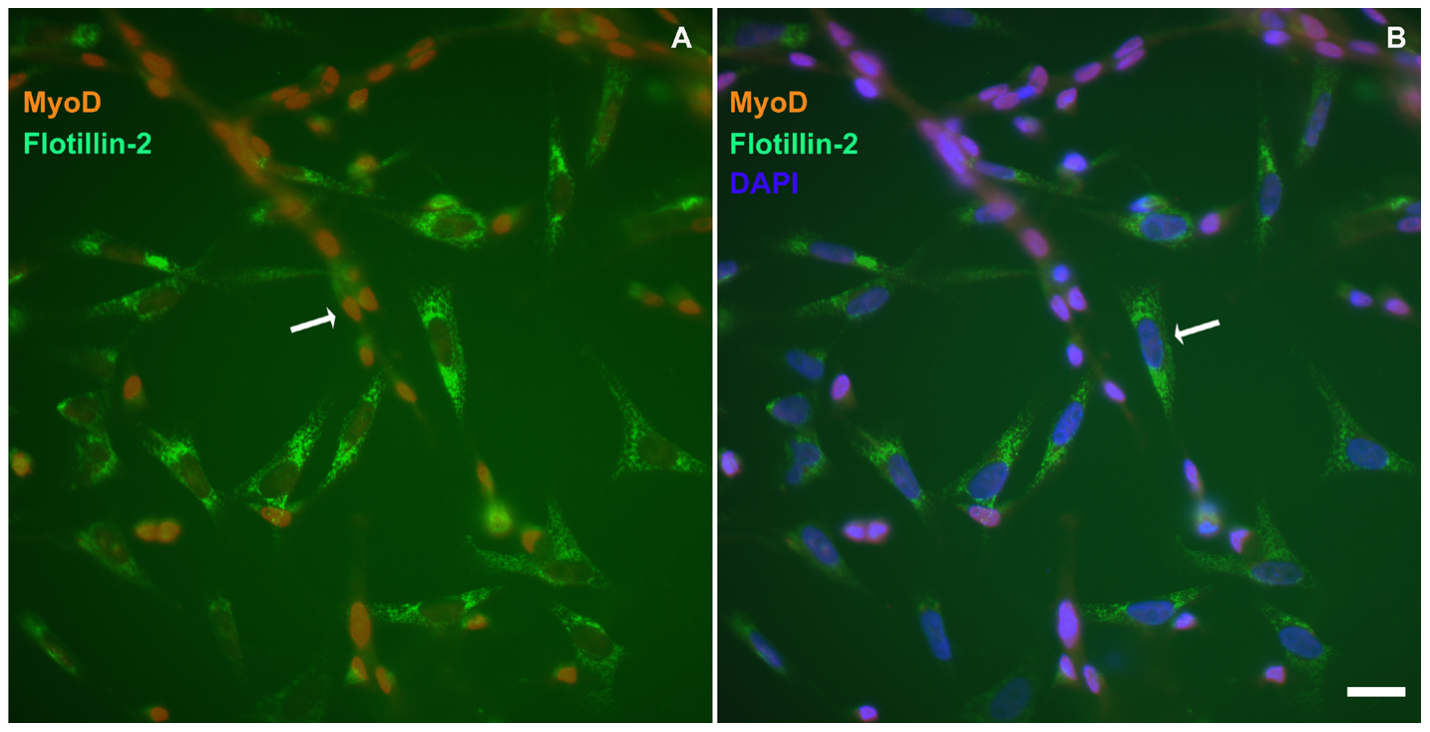
Flotillin-2 is mainly expressed in fibroblasts and weakly expressed in myoblasts. Myogenic cells were grown for 24(**A** and **B**). Cells were fixed with paraformaldehyde and stained with antibodies against MyoD (**red**, **A** and **B**) and flotillin-2 (**green**, **A**, and **B**) and with the nuclear dye DAPI (**blue**, **B**). Merged images are shown in **A** and **B**. Note that flotillin-2 is highly expressed in MyoD-negative cells and weakly expressed in MyoD-positive cells (**A** and **B**). Scale bar in **B** represents 20 µm.

To determine whether flotillin-2 positive cells were fibroblasts and/or myoblasts in chick myogenic cultures, we triple-labeled 24-h cells with an anti-chick MyoD antibody [10], an anti-flotillin-2 antibody, and the nuclear dye DAPI. The results show that MyoD-negative cells were highly labeled for flotillin-2, while MyoD-positive cells were weakly labeled for flotillin-2 (Figure 3). These data indicate that in chick myogenic cultures flotillin-2 is mainly expressed in fibroblasts and weakly expressed in myoblasts. We found that both round and bipolar MyoD-positive-myoblasts were weakly positive for flotillin-2. These data suggest that flotillin-2 is expressed in rounded chick myoblasts prior to their elongation and fusion, and that somehow flotillin-2 is redistributed and/or down-regulated after myoblast fusion into myotubes. These results are in agreement with the work of Volonté and co-workers [9], who showed that flotillin-2 is virtually absent from mature skeletal muscle tissues (diaphragm and psoas muscle) of mice. Draeger and colleagues [15] also showed that profound structural rearrangements occur during skeletal muscle maturation including a striking decrease in membrane lipid segregation and a down-regulation of raft-associated proteins.

**Figure 4:**
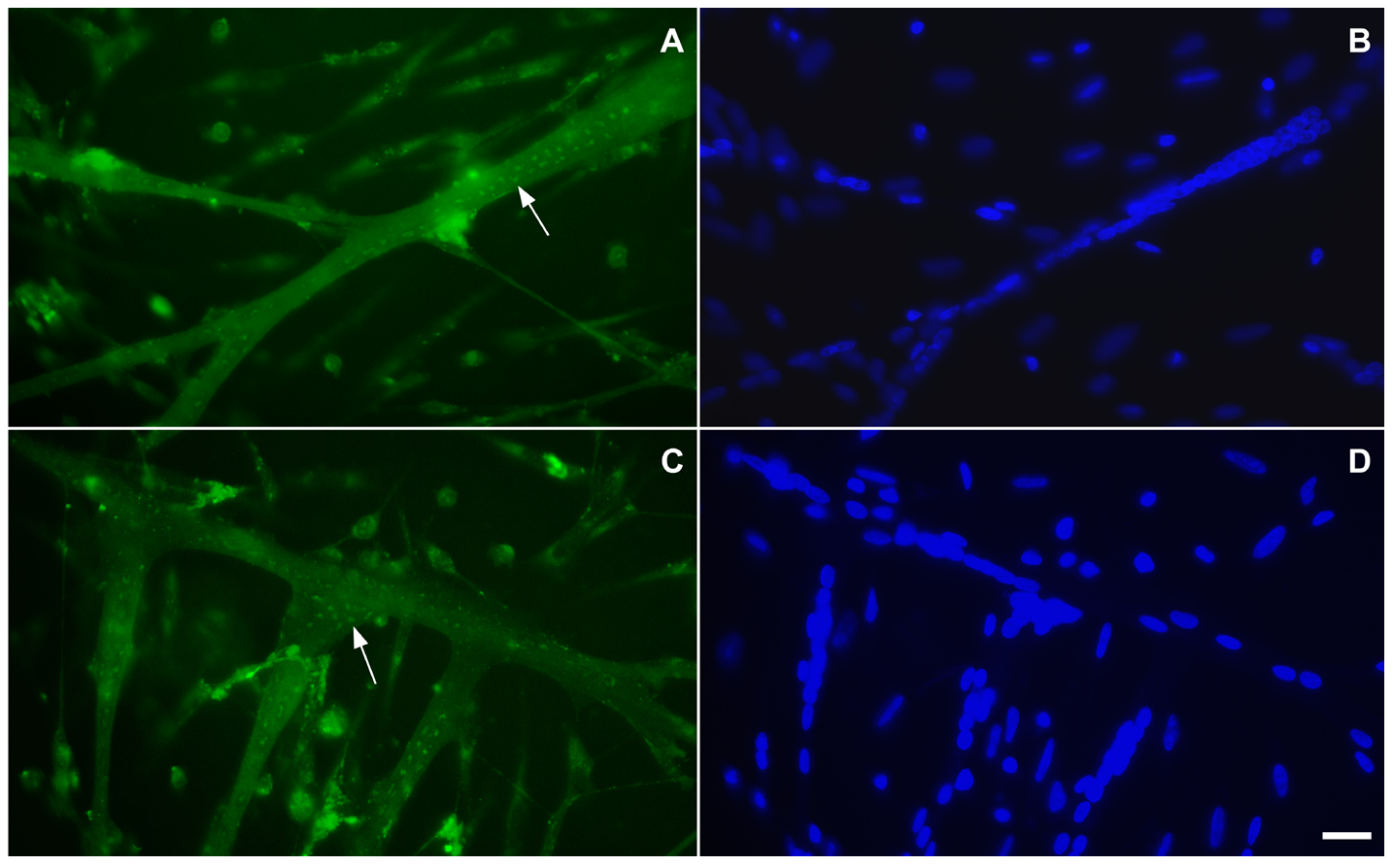
Flotillin-2 distribution in multinucleated myotubes. Myogenic cells were grown for 72-flotillin-2 antibodies (green, **A** and **C**) and the nuclear dye DAPI (blue, **B** and **D**). Note that with methanol fixation it is possible to see that flotillin-2 is present in elongated dots in myotubes (arrows in **A** and **C**). Scale bar in **D** represents 20 µm.

Since we found very little flotillin-2 staining in myotubes after paraformaldehyde fixation (Figures 2 and 3), we decided to rule out the possibility of fixation artifacts by testing other fixation protocols. Methanol fixation showed flotillin-2 concentrated in elongated dots in the subsarcolemmal region of 72-h chick multinucleated myotubes (Figure 4).

**Figure 5:**
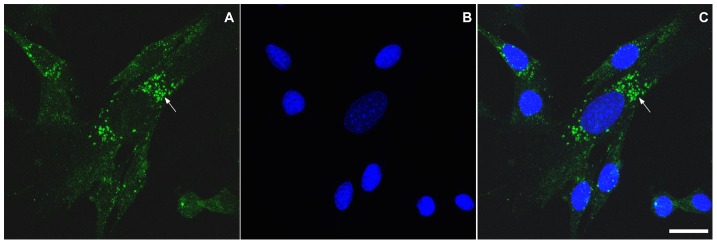
Distribution of flotillin-2 in C2C12 mouse muscle cell line. C2C12 cells were grown as described in Materials and Methods section. Cells were fixed with methanol and stained with an anti-flotillin-2 antibody (**green**, **A** and **C**) and the nuclear dye DAPI (**blue**, **B** and **C**). A merged image is shown in **C**. Note that flotillin-2 is present in C2C12 cells in vesicle-like structures (arrows in **A** and **C**). Scale bar in **C** represents 20 µm.

**Figure 6:**
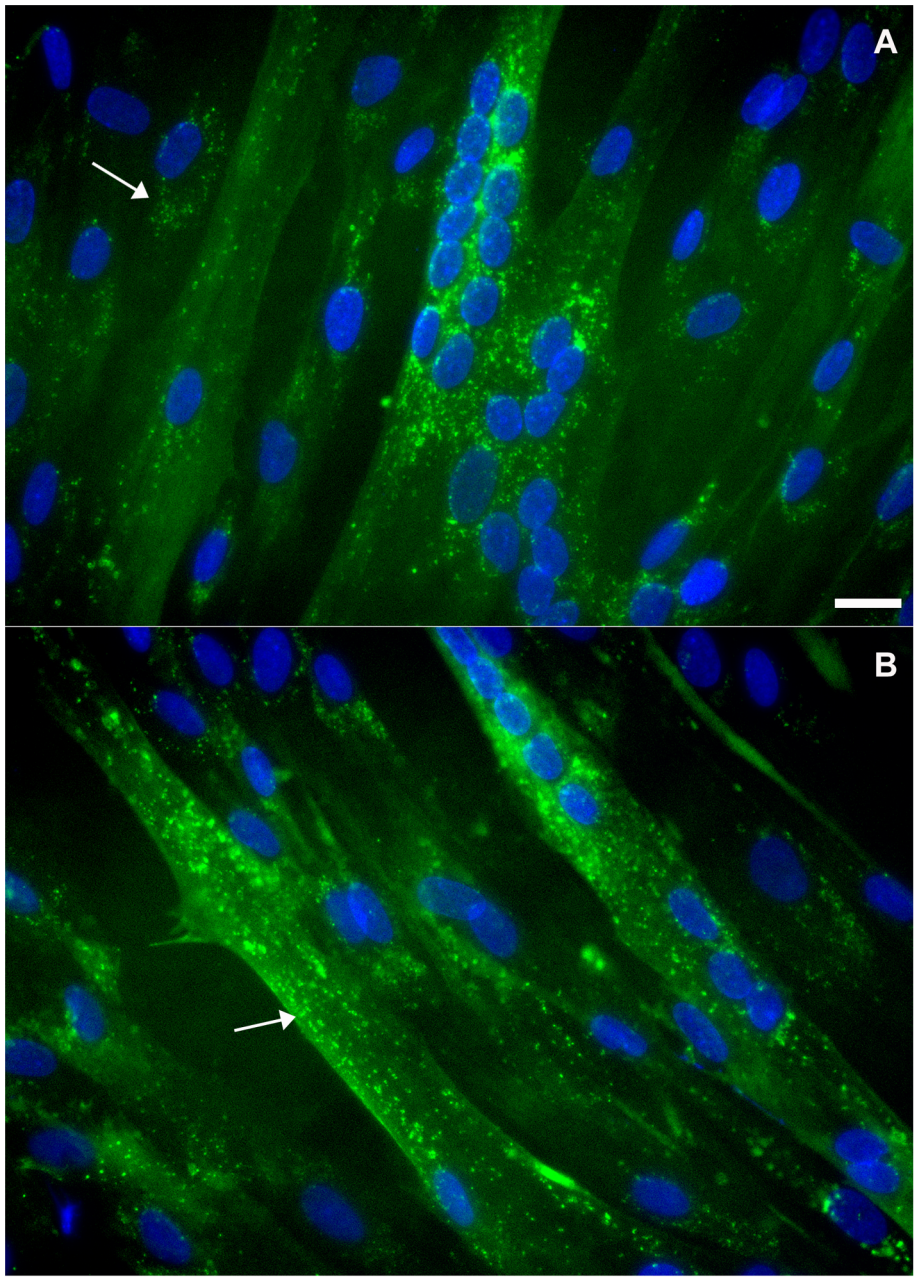
Distribution of flotillin-2 in human neonatal muscle cells. Human neonatal muscle cells were grown as described in Materials and Methods section. Cells were fixed with methanol and stained with an anti-flotillin-2 antibody (**green**, **A** and **B**) and the nuclear dye DAPI (**blue**, **A** and **B**). Merged images are shown in **A** and **B**. Note that flotillin-2 is present in human myoblasts and myotubes in vesicle-like structures (arrows in **A** and **B**). Scale bar in **A** represents 10 µm.

Interestingly, flotillin-2 staining was found in vesicle-like structures within the cytoplasm of chick fibroblastic cells (Figures 2 and 3). Our data are consistent with the work published by Langhorst and colleagues [6] where they show that flotillin-2 is present in vesicles in subconfluent HeLa cell cultures. Flotillin-2 has been identified in different cellular compartments, including lipid rafts at the plasma membrane, lipid droplets [7], and vesicles of the endocytic pathway [8]. Flotillin-2 distribution varies among different cell types. Thus we next compared the distribution of flotillin-2 in chick muscle cells with that in human and mouse muscle cells. We labeled mouse myogenic cell line C2C12 and neonatal human muscle cells with flotillin-2 using both paraformaldehyde and methanol fixation. No flotillin-2 labeling was found after paraformaldehyde fixation in the mouse and human muscle cells, but a positive labeling was found after methanol fixation. Control experiments with no primary antibodies showed only a faint background staining in both mouse and human cells (data not shown). After methanol fixation, C2C12 myoblasts and neonatal human muscle cells showed flotillin-2 in a vesicle-like labeling (Figures 5 and 6). Flotillin-2 staining in the mouse and human muscle cells was similar to the distribution we found in chick fibroblasts (Figures 2 and 3). It is important to point out that we found differences in flotillin-2 distribution between chick primary myoblasts and mouse C2C12 myoblasts (compare Figures 2 and 5). Flotillin-2 was densely present in cytoplasmic vesicles in C2C12 cells, whereas little flotillin-2 labeling was found in chick primary myoblasts. Interestingly, Banning and colleagues [16] state that they were unable to find a single cell line that did not express flotillins, indicating that they may be essential for cultured cell lines. These results open an interesting question related to differences between myoblast cell lines and myoblast primary cultures. It is possible to speculate that flotillin-2 is preferentially expressed in proliferative cells.

To better characterize the flotillin-2 positive vesicles, we measured their diameter in C2C12 cells, neonatal human muscle cells and chick fibroblasts. Quantification using the Fiji software (based on ImageJ, http://imageJ.nih.gov/ij/) showed that vesicles from the three cell types had a diameter in the range of 0.2–0.6 µm.

It is important to point out that we tested two different protocols for the fixation of cells prior to flotillin-2 labeling. We used a paraformaldehyde and a methanol fixation in order to see possible differences in the labeling of flotillin-2 with these two fixatives. Interestingly, chick fibroblasts were preferentially labeled with the anti-flotillin-2 monoclonal antibody after paraformaldehyde fixation, while after methanol fixation preferentially labeling occurred in chick multinucleated myotubes, mouse C2C12 cells and human neonatal muscle cells. These results show that fixation protocols for flotillin-2 immunolabeling can vary among different cell types and care should be taken when studying flotillin distribution in any cell type.

**Figure 7:**
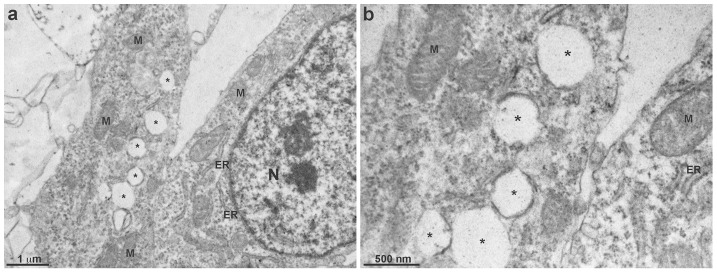
Transmission electron microscopy of chick fibroblastic cells. Chick myogenic cells were grown for 48(asterisks), well-preserved mitochondria (M), as well as several endoplasmic reticulum membranous profiles (ER) and a nucleus (N). Image shown in **b** is a higher magnification of a region of the image shown in **a**. Scale bar in **a** represents 1 µm and in **b** represents 500 *µ*m.

Since the immunofluorescence images of chick fibroblastic cells showed a high density of vesicular structures (Figures 2 and 3), we decided to further analyze these vesicles under transmission electron microscopy (TEM, Figure 7). It is possible to see a large number of vesicles within the cytoplasm of chick fibroblastic cells (Figure 7, asterisks). Measurements of these vesicles observed under TEM showed an average diameter of 0.4 µm, in accordance with the quantification of the diameter of flotillin-2 positive vesicles from immunofluorescence images.

**Figure 8:**
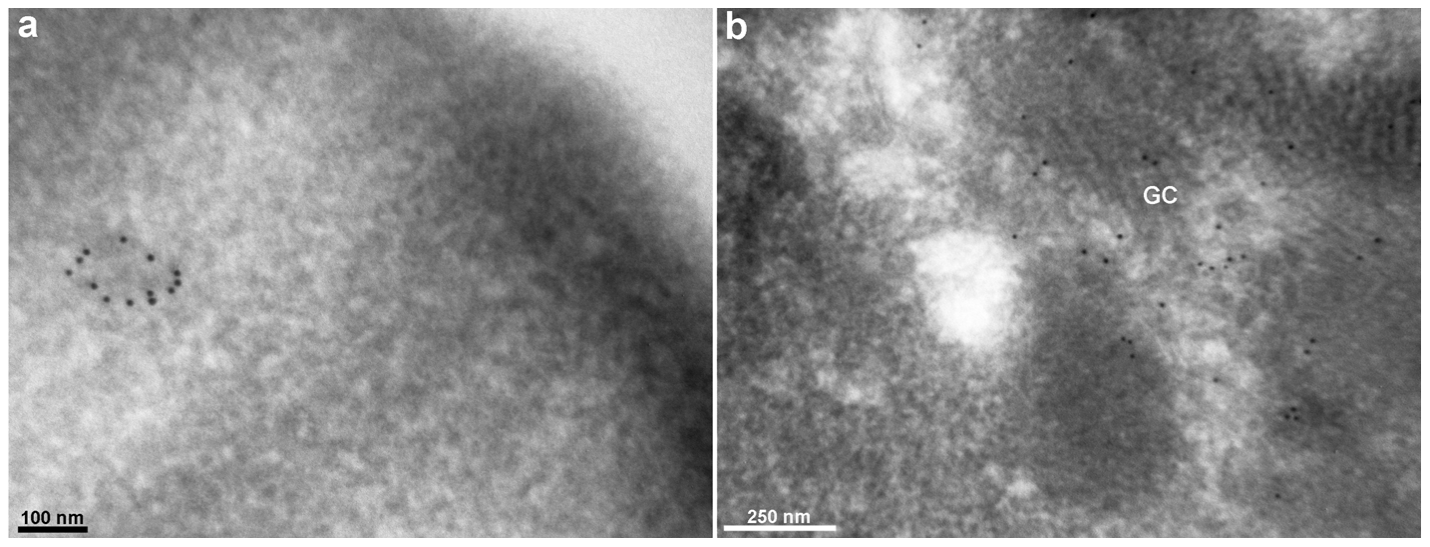
Cryo-immunogold EM labeling of flotillin-2 in myogenic cells. Chick myogenic cells were grown for 48-flotillin-2 antibody followed by a 10-*η*m gold-conjugated secondary antibody. Note that gold particles are present in vesicles (**a**) and associated with Golgi stacks (**b**). GC, Golgi complex. Scale bar in **a** represents 100 *η*m and in **b** represents 250 *η*m.

In order to confirm the presence of flotillin-2 in vesicular structures in chick fibroblasts, we studied the subcellular distribution of flotillin-2 after cryo-immunogold labeling in electron microscopy. Gold particles were found in the Golgi apparatus and in the membrane of vesicles found in the cytoplasm of chick fibroblastic cells (Figure 8). The presence of flotillin-2 in vesicles and in the Golgi suggests a role of flotillin-2 in the secretory pathway in chick muscle fibroblasts. Further studies are necessary to elucidate the possible involvement of flotillin-2-positive vesicles in the secretion of specific extracellular signaling molecules by fibroblastic cells. It is important to point out that we did not observe gold particles at the cell membrane of chick fibroblasts after the cryo-immunogold labeling in electron microscopy.

Flotillin-2 interacts with cholesterol in planar microdomains (non-caveolar rafts) at the plasma membrane. So, we decided to test whether cholesterol removal from the cell membrane could change the distribution of flotillin-2 in myogenic cells. Myogenic chick cell cultures were treated at 24 h with 2 mM of the cholesterol depleting drug methyl-β-cyclodextrin (MbCD) for 30 min, grown for the next 24 hours and immunolabeled for flotillin-2 and desmin. MbCD treatment induced a 15% increase in myoblast fusion, as analyzed by the quantification of the number of nuclei in myotubes divided by the total number of nuclei. Myoblast fusion was quantified in cells that were fixed with paraformaldehyde and double-labeled for desmin and DAPI.

**Figure 9:**
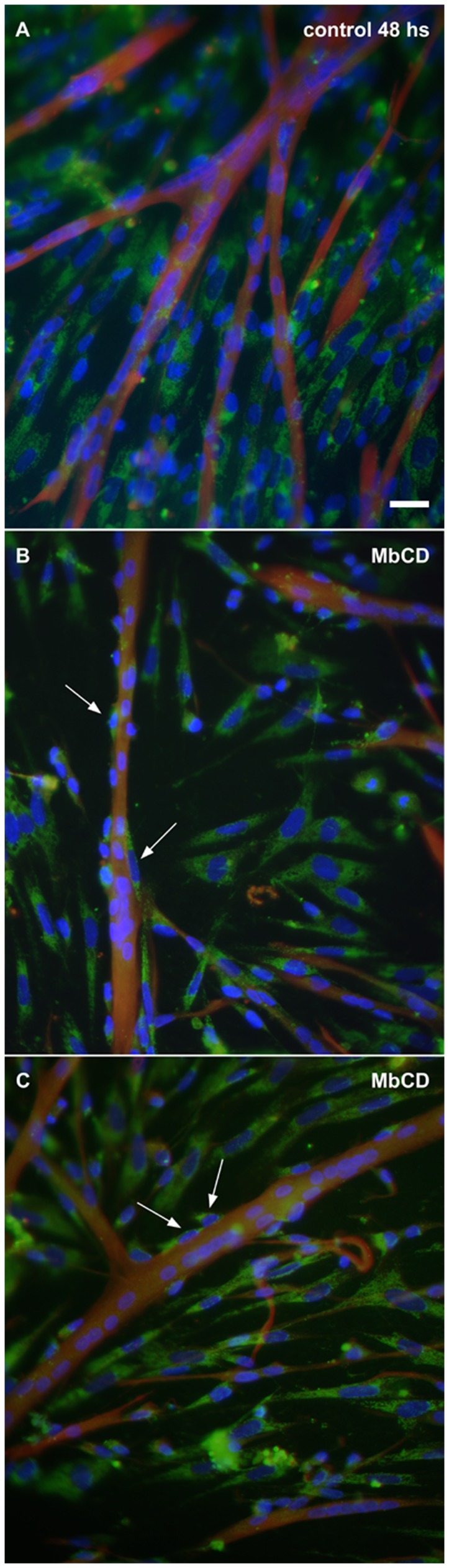
Flotillin-2 is found in pre-fusion myoblasts after cholesterol depletion. Immunofluorescence of flotillin-2, desmin and DAPI in cultures treated with methyl-β-cyclodextrin (MbCD) showing flotillin-2 expression in mononucleated cells that are fusing with myotubes. Chick myogenic cells were grown for 24 hours, treated with 2 mM MbCD for 30 min and grown for the next 24 hours (**B** and **C**). Some cells were not treated and were fixed at 48 hours of culture (control, **A**). All cells were fixed with paraformaldehyde and stained with antibodies against desmin (**red**) and flotillin-2 (**green**) and with the nuclear dye DAPI (**blue**). Merged images are shown in **A–C**. Note an increase in the number of flotillin-2 positive-mononucleated cells in close contact with the membrane of multinucleated myotubes (arrows in **B** and **C**). Scale bar in **A** represents 20 µm.

Interestingly, after cholesterol depletion it was possible to observe an increase in the number of flotillin-2-positive mononucleated cells in close contact with the cell membrane of multinucleated myotubes (Figure 9). Previously, our group has shown that cholesterol depletion by 2 mM MbCD induces an increase in myoblast proliferation and in myoblast fusion in chick primary cultures of skeletal muscle cells [2], [17]. It has been shown by Langhorst and colleagues [6] that cholesterol extraction by 12 mM MbCD reduces flotillin-2 vesicle trafficking in HeLa cells. Flotillins have been implicated in membrane trafficking and in the recruitment of specific molecules to cell contact sites. Regulated cycling of membrane proteins enables cells to rapidly modulate protein levels at the plasma membrane. Changes in the cycling rate of a vesicular pathway will immediately affect the distribution of its associated proteins between intracellular compartments and the plasma membrane [18].

**Figure 10:**
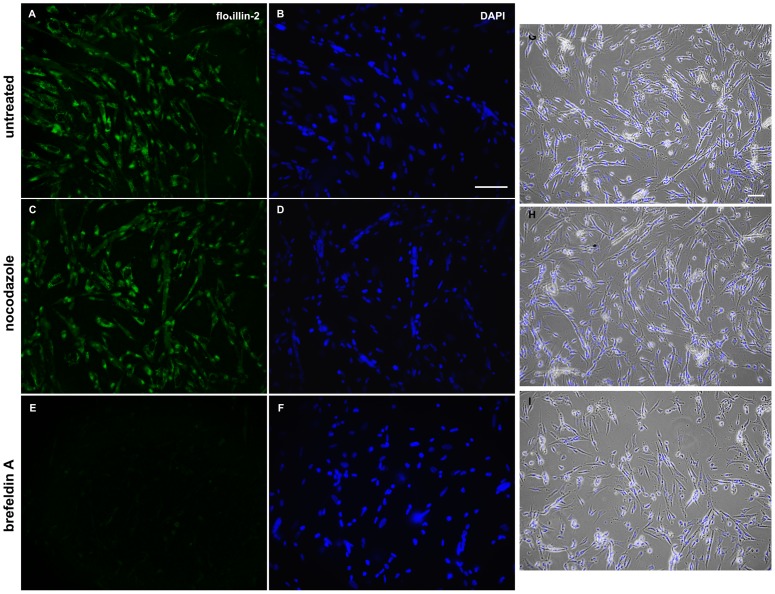
Brefeldin A induces a major reduction in the number of flotillin-2 containing vesicles. Chick myogenic cells were grown for 24(5 µg/ml) for 3 hours or with nocodazole (10 µg/ml) for 3 hours. After treatment, cells were grown in fresh culture medium for the next 3 hours. In images (**A–F**) cells were fixed with paraformaldehyde and stained with an antibody against flotillin-2 (**green, A,C,E**) and with the nuclear dye DAPI (**blue, B,D,F**). Note that while nocodazole have no effect in the presence and distribution of flotillin-2 positive vesicles (**C,D**), brefeldin A induced a major reduction in flotillin-2 containing vesicles (**E,F**). In images (**G–I**) cells were analyzed under phase contrast microscopy and superimposed with DAPI (blue). Scale bars represents 50 µm (in **A–F** and **G–I**).

In order to test the hypothesis that flotillin 2-containing vesicles somehow are involved in chick myoblast fusion, we try to disrupt intracellular vesicles with two agents that have been described to be able to disorganize intracellular vesicles: brefeldin A and nocodazole (Figure 10). Our results show that while nocodazole have no effect in the presence and distribution of flotillin-2 positive vesicles, brefeldin A induced a major reduction in the number of flotillin-2 containing vesicles (Figure 10). Quantification of myoblast fusion index showed a 13% decrease in chick myoblast fusion after brefeldin A treatment (Table 1). The fungal macrocyclic lactone brefeldin A has been shown to inhibit protein trafficking in the endomembrane system of eukariotic cells [19], by specifically blocking the intracellular transport of protein from endoplasmic reticulum to the Golgi apparatus. In agreement with our data, Ichikawa and colleagues [20] described that brefeldin A inhibited myotube formation in the mouse C2C12 cell line. We can conclude from our data that brefeldin A treatment is related both to a decrease in the number of flotillin-2 containing vesicles and to a decrease in myoblast fusion in chick myogenic cultures.

**Table 1:** Quantification of myoblast fusion after nocodazole and brefeldin A treatment.

control	80±3
nocodazole	76±4^n.s.^
brefeldin A	67±3*

Myoblast fusion index (the number of nuclei in myotubes divided by the total number of nuclei) was quantified in untreated and treated chick myogenic cultures. Cells were fixed with paraformaldehyde and double-labeled for desmin and DAPI. Nuclei were counted in fifty randomly chosen microscope fields (3 culture dishes for each of the experimental condition, 3 fields in each dish). Data are expressed as percentage of myoblast fusion (mean ± SEM). n.s. = not significantly different from control.

*p<0.05; t test for unpaired samples.

**Figure 11:**
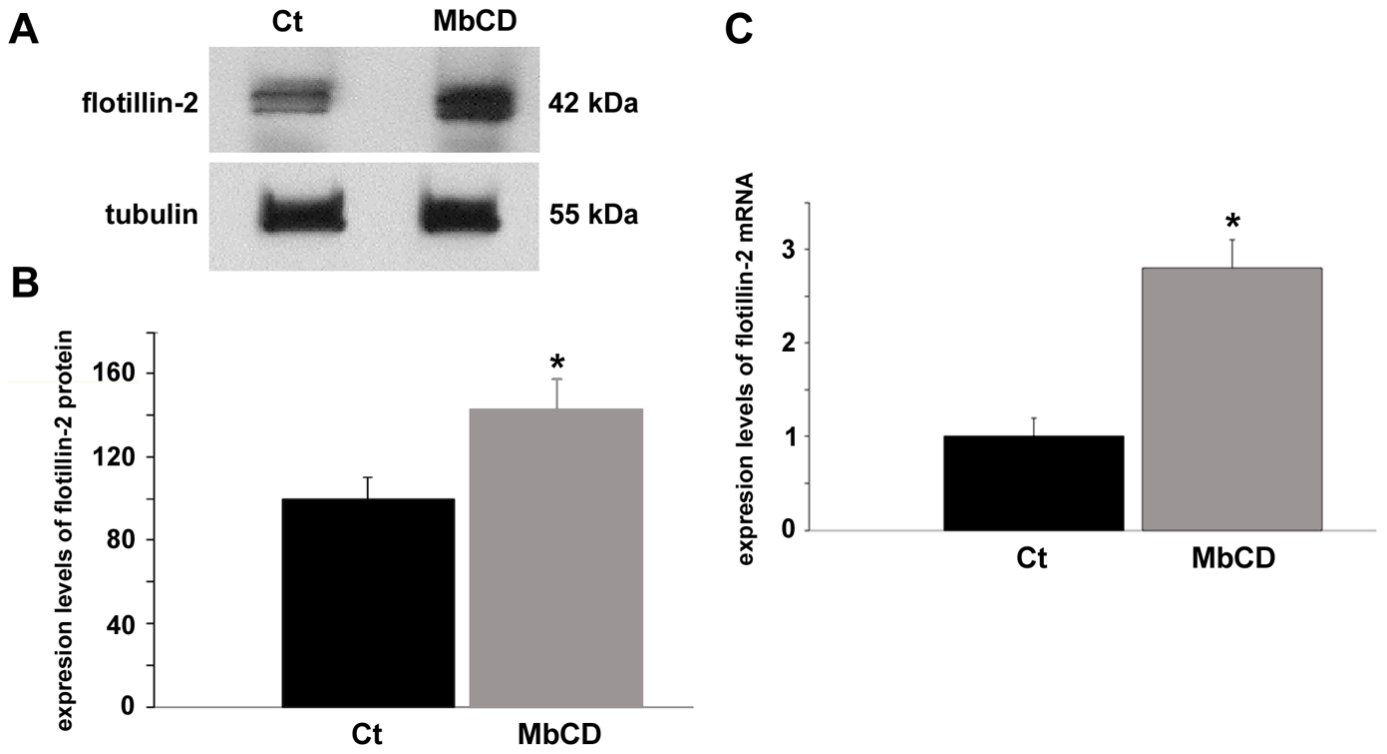
Cholesterol depletion enhances the expression of flotillin-2 protein and mRNA. Chick myogenic cells were grown 24(control, **Ct**). Cell culture extracts were analyzed in Western blot using an antibody against flotillin-2 (**A**). Lower Western blot shows α-tubulin reactivity of the same samples, and was used to normalize sample loading (**A**). Quantification of protein bands revealed a 40% increase in the levels of flotillin-2 expression after cholesterol depletion (**B**). RT-PCR analysis (for details, see Materials and Methods) of the expression of flotillin-2 in control and in MbCD-treated cells is shown in **C**. Glyceraldehyde-3-phosphate dehydrogenase (GAPDH) was used for normalization. Analysis of the expression of flotillin-2 shows a more than 2-fold increase in the levels of mRNA expression in MbCD-treated cells compared with control cells. *p<0.05; t test for unpaired samples, n = 3.

Since we found an increase in the number of flotillin-2 positive mononucleated cells after cholesterol depletion (Figure 9), we confirmed these data with the analysis of the expression of flotillin-2 mRNA and protein in chick myogenic cells. Chick myogenic cell cultures were treated at 24 h with 2 mM MbCD for 30 min, grown for the next 24 hours and subjected to a Western blot against flotillin-2. Results showed that MbCD induced a 40% increase in the expression of flotillin-2 protein as compared to untreated cells (Figure 11). We also analyzed the expression levels of flotillin-2 transcripts in control and MbCD-treated cells. Real time-PCR data revealed a more than 2-fold increase in the levels of flotillin-2 mRNA after cholesterol depletion (Figure 11). These results are in agreement with the data presented in Figure 9 showing that cholesterol depletion induced an increase in the number of flotillin-2-positive cells.

In conclusion, the results presented here demonstrate that: (1) flotillin-2 is down-regulated during chick *in vitro* myogenesis; (2) flotillin-2 is predominantly expressed in chick fibroblastic cells, while it is weakly expressed in chick myoblasts and in multinucleated myotubes; (3) flotillin-2 is present in vesicle-like structures and in the Golgi in chick fibroblasts, in the mouse C2C12 myogenic cell line and in human neonatal muscle cells; (4) cholesterol depletion induces an increase in the expression of flotillin-2, at the levels of both mRNA and protein, in chick myogenic cells; and (5) disruption of flotillin-2-positive vesicles with brefeldin A induces a decrease in myoblast fusion.

The present work points to new functions of flotillin-2 in the initial steps of chick skeletal muscle differentiation, such as myoblast migration, cell recognition and fusion. Further, flotillin-2 might be used as a marker for fibroblasts in chick skeletal muscle cultures, since it is highly expressed in fibroblasts and weakly expressed in myoblasts and multinucleated myotubes. These results also raise an interesting question related to the biological meaning of the differences between the expression of flotillin-2 in chick fibroblasts and chick muscle cells (myoblasts and myotubes).
